# Predicting the potentially exacerbation of severe viral pneumonia in hospital by MuLBSTA score joint CD4 + and CD8 +T cell counts: construction and verification of risk warning model

**DOI:** 10.1186/s12890-024-03073-y

**Published:** 2024-05-29

**Authors:** Xi Chen, Bei Ma, Yu Yang, Mu Zhang, Fang Xu

**Affiliations:** 1 Department of Critical Care Medicine, The First Affiliated Hospital of Chongqing Medical University, No.1 Youyi Road, Yuzhong District, Chongqing, 400016 China; 2 Department of Critical Care Medicine, People’s Hospital of Chongqing Liangjiang New Area, Chongqing, 401120 China

**Keywords:** Viral pneumonia, MuLBSTA score, Warning system

## Abstract

**Purpose:**

This study mainly focuses on the immune function and introduces CD4^+^, CD8^+^ T cells and their ratios based on the MuLBSTA score, a previous viral pneumonia mortality risk warning model, to construct an early warning model of severe viral pneumonia risk.

**Methods:**

A retrospective single-center observational study was operated from January 2021 to December 2022 at the People's Hospital of Liangjiang New Area, Chongqing, China. A total of 138 patients who met the criteria for viral pneumonia in hospital were selected and their data, including demographic data, comorbidities, laboratory results, CT scans, immunologic and pathogenic tests, treatment regimens, and clinical outcomes, were collected and statistically analyzed.

**Results:**

Forty-one patients (29.7%) developed severe or critical illness. A viral pneumonia severe risk warning model was successfully constructed, including eight parameters: age, bacterial coinfection, CD4^+^, CD4^+^/CD8^+^, multiple lung lobe infiltrations, smoking, hypertension, and hospital admission days. The risk score for severe illness in patients was set at 600 points. The model had good predictive performance (AUROC = 0.94397), better than the original MuLBSTA score (AUROC = 0.8241).

**Conclusion:**

A warning system constructed based on immune function has a good warning effect on the risk of severe conversion in patients with viral pneumonia.

**Supplementary Information:**

The online version contains supplementary material available at 10.1186/s12890-024-03073-y.

Viral pneumonia is an important cause of severe pneumonia in adults [1], often accompanied by acute respiratory distress syndrome (ARDS), which prolong the course of the disease, elevated the mortality rate, and increase the treatment cost [2, 3]. Even though oseltamivir (for influenza A) and Nirmatrelvir/Ritonavir (for COVID-19) can play the antiviral role, there are still many problems, such as the dependence on the detection of pathogenic microorganisms in the early stage, the control of indications, etc. Moreover, in some cases, it cannot block the immune escape, cytokine storms, and persistent lung injury induced by viral infection. Early identification of patients with a tendency to develop severe illness and actively blocking their progression is a key link in reducing the mortality rate of viral pneumonia [4]. As hypoxia is a crucial pathophysiological occurrence in viral pneumonia, providing early noninvasive support to enhance oxygenation and protect the lungs and diaphragm may prevent tracheal intubation, thus avoiding complications of sedation and invasive mechanical ventilation [5]. And there are also new studies focus on anti-inflammatory therapies, such as blocking NF-κB and activate Nrf2 pathways [6].The MuLBSTA score, first proposed in 2019 by a team led by Professor Min Zhou and Professor Jieming Qu from the Department of Respiratory and Critical Care Medicine at Shanghai Ruijin Hospital in China, has a high value in predicting the risk of death from viral pneumonia and includes six common parameters: age, smoking status, hypertension, multiple lobar infiltration, lymphocyte ≤ 0.8 ∗ 10^9^ /L, and bacterial coinfection [7]. Peripheral blood T-lymphocytopenia may be the main cause of lymphocytopenia in severe viral pneumonia, although the exact cause is not fully understood [8]. T lymphocytes play a crucial role in adaptive immune protection [9]. CD4^+^ T cells secrete cytokines to support the function of CD8 T cells and B cells, while CD8^+^ T cells eliminate viruses by killing infected cells [10]. Severe SARS-CoV-2 infection leads to T-cell dysregulation, excessive T-cell-mediated cytokine release, and widespread systemic inflammation and tissue damage. Inhibition of host-derived Dkk-1 can restore T cell distribution, enhance SARS-CoV-2 virus clearance, and reduce lung injury [11]。Under certain inflammatory conditions, regulatory T (iTreg) cells may lose FoxP3 expression and acquire a pro-inflammatory cytokine-expressing pathogenic phenotype. The protein pp6, which acts as a key positive regulator of FoxP3, helps to maintain Treg cell stability and prevent immune dysregulation. These findings suggest that T cell function may play an important role in the development of inflammatory diseases [12]. Single-cell transcriptome analyses showed there were apoptosis in various CD4^+^T cells and CD8^+^T cells subsets [13, 14], and some special effect subgroups were associated with convalescence in clinical moderate groups, after respiratory viruses infection(H1N1)[14]and COVID-19 [15]. Therefore CD4^+^T cells and CD8^+^T cells should be the immune function indicators to promote predictive performance of MuLBSTA score. In order to investigate the above clinical scenario, we established a retrospective single-center research cohort and build a more convenient model to predict the incidence of severe viral pneumonia, providing a basis for early treatment planning and prognostic evaluation.

## Materials and methods

### Study design

A retrospective single-center observational study was conducted at the People's Hospital of Liangjiang New Area, Chongqing, China, from January 2021 to December 2022. The study was approved by the hospital's ethics committee. We retrospectively analyzed hospitalized patients who tested positive for respiratory viruses using a multiplex real-time reverse transcription-polymerase chain reaction assay. Patients in hospital who were diagnosed pneumonia according to the 2009 Infectious Diseases Society of American (IDSA)/American Thoracic Society (ATS) guidelines were enrolled in this study [16]. Patients were excluded if: 1) under 18 years old, had liver function failure;2) were long-term dialysis patients; 3) had active tuberculosis; 4) had thrombosis or pulmonary embolism; 5) had primary immunodeficiency diseases or were receiving immunosuppressive therapy (such as AIDS, post-organ transplantation or neutropenia); 6) were undergoing anti-tumor drug therapy or radiation therapy; 7) were unable to undergo the required experimental investigations. A total of 235 hospitalized patients tested positive for viral infection, among whom 162 cases were diagnosed as pneumonia. A total of 24 patients were excluded:19 patients had other diseases affecting immune function, and 5 patients lacked relevant laboratory tests. Other exclusion criteria excluded no patients from the study, so exclusion criteria were 0. Finally, 138 patients with pneumonia who tested positive for the virus were enrolled (Supplement Fig. 1).

### Data collection

Various viruses, including influenza (Flu), coronavirus (CoV), adenovirus (AdV), bocavirus, human rhinovirus (HRV), respiratory syncytial virus, Human parvovirus parainfluenza virus (PIV), enterovirus (EV), and human metapneumovirus (HMPV), were Validated through RT-PCR. Information was collected upon admission, including demographic data, comorbidities, CCI (Charlson Comorbidity Index) [17], laboratory results, CT scans, etiological and immunological testing. Positive bacterial cultures from blood or sputum specimens were used as a criterion for co-infection with bacteria, and antiviral and steroid regimens were recorded. Viral pneumonia patients were divided into severe and non-severe groups, with the severe group including critically ill and severely critically ill patients. Critically ill patients present one of the following symptoms: (1) Dyspnea (> 30 breaths/minute), (2) resting oxygen saturation < 93%, or (3) oxygenation index (PaO2/FiO2) < 300 mmHg; Severely critically ill patients exhibited any of the following: (1) respiratory failure requiring invasive ventilation, (2) shock, or (3) other organ failures requiring ICU care. The MuLBSTA score was counted as deemed clinically appropriate at any time when pneumonia was suspected, and the hospital admission time, hospitalisation time and prognosis were recorded. The result of the mortality rate was defined as the total mortality rate within 90 days.

### Statistical analysis

Normality was tested using the Shapiro–Wilk normality test statistic; normally distributed data was denoted by ‾x ± s; intergroup comparisons were performed using t-tests. The comparison of unordered count data between groups was performed using the chi-square test or Fisher's exact test. The division point was determined based on the Youden's index of the receiver operating characteristic (ROC) curve or clinically relevant cutoff points. Variables with *p* < 0.10 were considered potential risk factors and included in the multivariate regression analysis. The predictive performance of the score was assessed by measuring the area under the ROC curve (AUROC). Statistical analyses were performed using SPSS version 22.0 and R 3.5. All tests were two-sided, and *p* values < 0.05 were considered statistically significant.

## Result

### Description of demographic characteristics

The baseline characteristics of the complete case are shown in Table 1. The average age of the patients with viral pneumonia in this study was 64.40 ± 16.43 years, with the majority being elderly, of which 60.14% were male. There were 41 patients with severe or critical illness, accounting for 29.7%. Nearly half of the patients had a history of smoking (49.27%, of which 10.14% had quit smoking before the onset of the disease). A total of 78 (56.52%) patients suffered from comorbidities, of which hypertension was the most common, accounting for approximately 36.96%, followed by diabetes, nephropathy, coronary heart disease, and chronic obstructive pulmonary disease (COPD). The virus types of the patients with viral pneumonia were shown in supplementary Table 1, which mainly consisted of novel coronavirus pneumonia. Multiple logistic regression analysis suggested that type of virus had no significant effect on risk of severe viral pneumonia(*P* = 0.785 > 0.05). Among the patients with viral pneumonia, 50 patients (36.23%) were co-infected with bacteria. The types of bacteria were shown in supplementary Table 2, among which Klebsiella pneumoniae, Acinetobacter baumannii and Escherichia coli were the three most common pathogens. Of all patients, 95 (68.84%) received antiviral treatment, 41 received high-flow/non-invasive ventilation, and 12 received invasive ventilation. There were statistically significant differences in age, smoking, hypertension, CCI, ICU admission days, hospital admission days, CD4^+^, CD8^+^, CD4^+^/CD8^+^, lymphocyte count, multiple lung lobe infiltrations, and bacterial coinfection, MuLBSTA between severe and non-severe groups of viral pneumonia. Subgroup analysis revealed significant differences in MulBSTA scores between admission and symptom deterioration among patients in the severe group (*P* < 0.0001) (supplement Fig. 2).

**Table 1:** Population description and comparison

	Total(*N* = 138)	No-Severe(*N* = 97)	Severe(*N* = 41)	p
Gender/Male	83(60.14%)	56(57.73%)	27(65.85%)	0.4264
Age	64.40 ± 16.43	59.98 ± 15.63	74.63 ± 13.38	0.0001
Acute-smoker	54(39.13%)	32(32.99%)	22(53.666%)	0.0378
Quit-smoker	14(10.14%)	9(9.28%)	5(12.20%)	
Non-smoker	70(50.72%)	56(57.73%)	14(34.15%)	
Comorbidity				
CCIs	3(1, 4)	2(1,3)	5(3,6)	< 0.0001
Hypertension	51(36.96%)	28(28.87%)	23(56.10%)	0.0025
COPD	24(17.39%)	16(16.49%)	8(19.51)	0.8062
Diabetes	15(10.87%)	9(9.28%)	6(14.63%)	0.3778
Coronary heart disease	8(5.78%)	5(5.15%)	3(7.32%)	0.6946
Cerebral infarction	7(5.07%)	3(3.09%)	4(9.76%)	0.1958
Renal disease	5(3.26%)	3(3.09%)	2(4.87%)	0.6335
History of malignancy	4(2.90%)	1(10.31%)	3(7.32%)	0.0785
Hepatitis	2(1.45%)	0	2(4.87%)	0.0867
Treatment				
High flow/noninvasive	41(29.71%)	12(12.37%)	29(70.73%)	
Invasive mechanical ventilation	12(8.79%)	0	12(29.27%)	
Antiviral treatment	95(68.84%)	60(68.04%)	29(70.73%)	
Steroid treatment	60(43.48%)	35(36.08%)	25(60.96%)	
Antibiotic therapy	59(42.75%)	27(27.84%)	32(78.05%)	
Vasoactive agent	15(10.87%)	0	15(36.59%)	
ICU days	2.405 ± 5.81	0	8.09 ± 8.279	< 0.0001
Hospitalization time	17.86 ± 34.8	11.71 ± 5.383	32.39 ± 61.15	0.0012
Hospital admission days	3.11 ± 3.37	2.71 ± 1.84	4.05 ± 5.42	0.032
CD4 +	414.4 ± 247.9	449.8 ± 233.5	283.7 ± 192.2	< 0.0001
CD8 +	261.6 ± 131.3	308.6 ± 165.8	245.7 ± 111.4	0.0242
CD4/CD8	1.672 ± 0.8903	1.865 ± 0.866	1.207 ± 0.756	< 0.0001
Lymphocyte count	1.073 ± 0.758	1.167 ± 0.66	0.858 ± 0.920	< 0.0001
Multilobar infiltrates	46(33.33%)	22(22.68%)	24(58.54%)	0.0024
Co-infection with bacteria	50(36.23%)	21(24.13%)	29(70.73%)	0.0002
MuLBSTA	7(4,11.75)	5(3,9)	12(9,16)	< 0.0001

*P*-value represented the comparison between Severe group and non-severe group. *p*-values < 0.05, indicated signifificant difference between Severe group and non-severe group. Hospital admission days: the time from admission to diagnosis of viral pneumonia and start of scoring. Hospitalisation time: the total time between admission and start of treatment and discharge. *COPD* Chronic obstructive pulmonary disease, *CCI* Charlson Comorbidity Index

### Determination of early warning model indicators

**Fig. 1:**
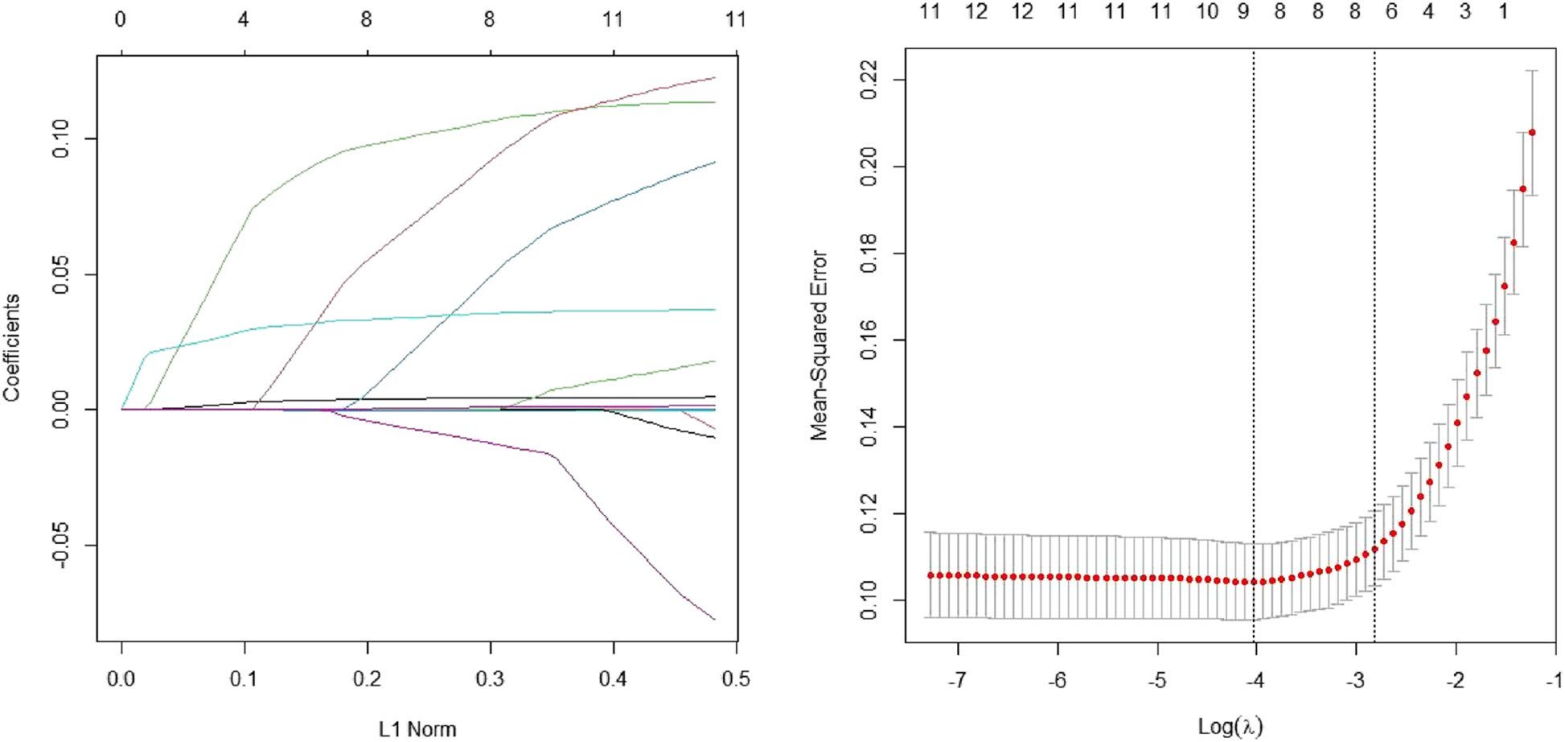
LASSO regression analysis: 9 features were identified as the potential predictors

Meaningful indicators from preliminary statistical analysis were selected using LASSO regression analysis and logistic multiple regression analysis to screen the model's independent variables. LASSO regression analysis was performed using R code, and as shown in Fig. 1, the dashed line on the right corresponds to a parameter of 9, indicating that the coefficients of 9 genes can be used as model parameters. The specific coefficient estimates for each independent variable obtained from the LASSO regression analysis are shown in supplementary Table 3. Among them, the independent variables with non-zero coefficient estimates are the selected independent variables, including age, Coinfection, CD4^+^, CD4^+^/CD8^+^ ratio, multiple lung infiltrates, smoking, hypertension, ICU admission days, and hospital admission days. The independent variables with larger coefficient estimates correspond to more important indicators, while those with smaller coefficient estimates can be screened out. Logistic multiple regression was then performed using R language, with severity as the independent variable for this prediction model. The analysis results are shown in supplementary Table 4: except for ICU admission days, there were significant differences (*P* < 0.05) between the other variables and the target variable (severity), including age, coinfection, CD4^+^, CD4^+^/CD8^+^, multiple lung infiltrates, smoking, hypertension, and hospital admission days, which were included in the model construction.

### Building a prediction model and evaluating its performance

**Fig. 2:**
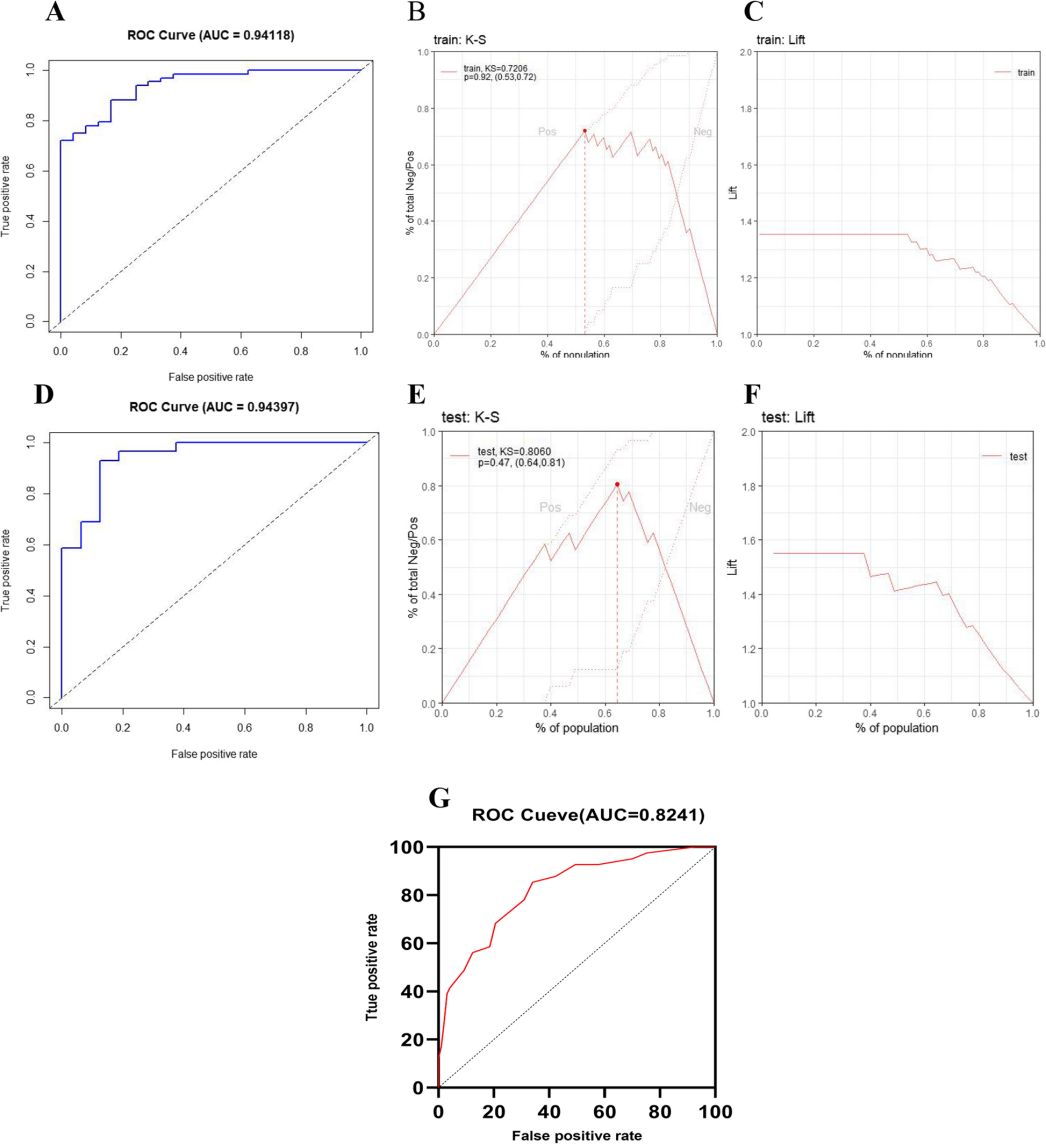
Building a Prediction Model and Evaluating its Performance. **A** ROC diagram of the training set model; **B **K-S diagram of the training set model; **C **Lift diagram of the training set model. **D **ROC diagram of test set model; **E** K-S diagram of test set model; **F **Lift diagram of test set model; **G **ROC curve of MulBSTA score prediction

In this study, we chose to use 70% of the original data as the training set and the 30% as the test set. The model prediction results of the training set and test set were evaluated using ROC value, K-S chart, and Lift chart. As shown in Fig. 2, the model result ROC of the training set data is 0.94118(Fig. 2A), the K-S curve is more obvious towards the upper left (Fig. 2B), and the Lift chart curve is relatively flat (Fig. 2C), indicating that the model has high accuracy in predicting severe cases. The test set model prediction results are shown in Fig. 2, with a ROC of 0.94397(Fig. 2D), which is better than the ROC of the training set model prediction results. The K-S curve is more obvious towards the upper left (Fig. 2E), and the Lift chart curve is relatively flat (Fig. 2F), indicating that the model prediction performance is stable and has high accuracy in predicting severe cases. Compared with the original MulBSTA score (Fig. 2G), this model has higher efficiency in predicting the risk of severe conversion (AUROC = 0.94397vs.0.0.8241).

### Production and evaluation of a severe risk scorecard

**Fig. 3:**
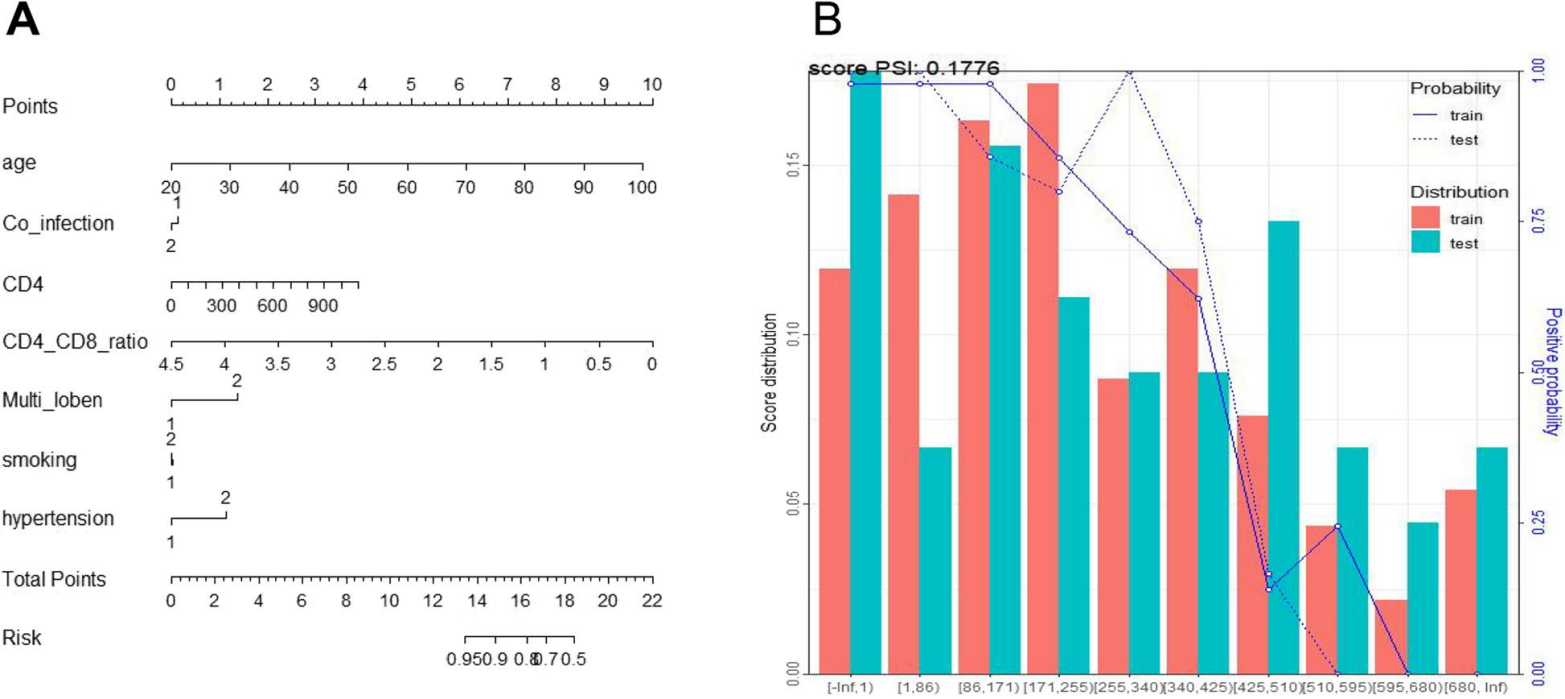
Evaluation of a severe risk scorecard. **A** Model nomogram; **B** Evaluation card PSI evaluation result plot

Based on the above analysis, the predictive model used this time has good predictive ability for whether patients will convert to severe cases. The evaluation card of the model is shown in Table 2. The score for whether the patient has a severe risk is set to 600 points. The visualization of the independent variables participating in the model is shown in Fig. 3A. Finaly this study used the PSI (Population Stability Index) index to evaluate the stability of the scorecard. The evaluation card indicators established in the model were calculated and visualized by PSI using R language, as shown in Fig. 3B. The PSI result for whether the patient has severe risk is 0.1776, which is less than 0.25, indicating that the changes are small and the model has good stability.

**Table 2:** Results of scorecard model bins

variate	Range	sample size	woe	Score
age	[-Inf,66)	40	1.156	-80
	[66,70)	15	1.598	-110
	[70,76)	16	-0.531	37
	[76,86)	12	-2.140	148
	[86, Inf)	9	-0.818	56
Co-infection	No	61	0.849	-60
	Yes	31	-1.106	78
CD4	[-Inf,140)	10	-1.889	42
	[140,180)	6	-1.041	23
	[180,340)	21	0.405	-9
	[340,360)	5	-1.447	32
	[360, Inf)	50	0.774	-17
CD4_CD8_ratio	[-Inf,0.9)	20	-1.447	76
	[0.9,1.6)	21	0.405	-21
	[1.6,1.7)	8	-0.531	28
	[1.7, Inf)	43	0.987	-52
Multi-lobe	No	63	0.512	-14
	Yes	29	-0.834	23
smoking	No	43	0.596	-32
	Once	9	0.211	-11
	Yes	40	-0.531	28
hypertension	[-Inf,2)	57	0.506	14
	[2, Inf)	35	-0.636	-18
Hospital admission days	[-Inf,8)	15	-0.348	23
	[8,12)	29	1.561	-101
	[12,16)	26	0.394	-26
	[16,22)	10	-1.447	94
	[22,32)	7	-0.125	8
	[32, Inf)	5	-2.428	158

## Discussion

Viral pneumonia is a lung inflammation caused by different types of viruses invading lung tissue and the incidence of viral pneumonia requiring ICU admission fluctuated between 16 and 49% [18]. Over the past two decades, 4 respiratory viruses have causing particular concern because of the high proportion of critically ill and acute respiratory failure among patients with viral infections: influenza, especially influenza A H1N1 2009; and 3 novel coronaviruses, Middle Eastern respiratory syndrome coronavirus, SARS coronavirus and severe acute respiratory syndrome coronavirus 2 (SARS-CoV-2) [19]. Although the survival rate of hospitalized patients with virus-associated pneumonia varies with the pathogenic virus subtype [20], ARDS, sepsis, hypercapnia associated with pulmonary fibrosis, and secondary bacterial infection [21] were the main causes for the process of becoming more serious. Virus infection will activate the host immune system. In severe cases, it can cause immune cells to secrete a large number of cytokines, trigger inflammatory storms that exacerbates immune disorders, coagulation dysfunction, and endothelial cell injury, leading to ARDS, sepsis, and multiple organ insufficiencies [22], resulting in higher ICU occupancy rates and mortality rates. Currently, clinical study on the novel coronavirus suggested that antiviral treatment should be initiated in a timely manner to delay disease progression and improve prognosis [23]. However, the diversity of virus types makes it difficult to standardize antiviral treatment plans. Therefore, there is an urgent need for clinicians to more accurately predict morbidity and mortality in critically ill patients with viral pneumonia [24].

In this study we found that immunocompetent status, age, and comorbidities were strongly associated with the severity of viral pneumonia. In fact, age is another independent risk factor for severe viral pneumonia [25]. The severity and risk of death increases significantly with age, especially in elderly patients over 65 years of age [26], partly due to an significantly immune responses of extreme age [27]. The Charlson Comorbidity Index (CCI) was proposed by Charlson et al. in 1987 to assess the impact of comorbidities on a patient's 10-year survival. The study found a statistically significant difference in the CCIs of patients with severe disease compared to those with non-severe disease. CCIs are of great significance for assessing the survival and prognosis of patients with severe disease. However, this study primarily focuses on the early warning signs of viral pneumonia in patients. In clinical research, the time of patient visit can vary greatly due to differences in disease onset cycles and self-perceived symptoms, which has always been a big problem in clinical research. Because we cannot predict hospitalization time at the diagnosis of viral infection, we chose the hospital admission days to study the risk of severe illness from viral pneumonia. The study discovered that the woe value of the diseased samples varied with the duration of hospital admission days. The inclusion of hospital admission days enables dynamic assessment of the risk score for critical illness.

MuLBSTA score is a multidimensional index score developed by Shanghai Ruijin Hospital to predict the risk of death from viral pneumonia, which includes age, smoking status, hypertension, multiple lobar infiltration, lymphocyte ≤ 0.8 ∗ 10^9^ /L, and bacterial coinfection [7]. Subsequent studies have also validated its usefulness in identifying high-risk patients with severe COVID-19 pneumonia [28, 29]. The ratio, number and function of different subpopulations of lymphocytes directly affect the immune status of the organism, and better reflect the immune function than the lymphocyte count, and helps to observe the therapeutic effect and evaluate the prognosis. Therefore, we introduced lymphocyte subpopulations, analyzed and compared the role of both in predicting viral pneumonia.

The pathogenesis of severe respiratory tract infections caused by viruses mainly involves the dysregulation of pro-inflammatory/anti-inflammatory responses and immune imbalance, such as overreaction of the immune system, excessive apoptosis and functional exhaustion of immune cells, immune suppression or immune paralysis. CD8^+^ and CD4^+^ T cells are essential for maintaining the balance between cellular and humoral immunity [30], and they are both involved in the deterioration of COVID-19 pneumonia. Studies showed that the changes in peripheral blood CD4^+^ and CD8^+^ T cells decreased significantly in severe and critical patients with COVID-19. The absolute value and percentage of CD8 + T cells showed a downward trend in ICU patients with viral pneumonia [31]. In this study, the number of CD4^+^ and CD8^+^ T cells in patients with severe and critical viral pneumonia were significantly decreased (Table 1). The ratio of CD4^+^ and CD8^+^ T cells represents the ratio of Th cells and suppressive T cells and the decrease in the ratio indicates a higher degree of immune aging and a higher risk of death [32]. However, this change was different after viral infection. In the critical patients of COVID-19, some researchers found that lymphocyte subsets were significantly decreased and CD4^+^/CD8^+^ T cell ratio remained normal [33]. However, in another group of COVID-19 infected patients, the CD4 + /CD8 + T cell ratio may also be elevated due to a more pronounced reduction in CD8^+^ T cells than in CD3^+^, CD4^+^ and CD8^+^ T lymphocytes [34]. There are also reports revealing the ratio of CD4^+^/CD8^+^ T cell decreased in viral pneumonia, which may be due to the suppression of CD4^+^ T cells, the decrease in the absolute number of lymphocytes, and the activation of cytotoxic CD8 + T cells [35]. Actually, different changes in CD4^+^/CD8^+^ ratio is a reflection to the immune function in different stages of viral pneumonia. This study introduced CD4^+^, CD8^+^ and their ratio, which could more stably and comprehensively reflect the patient's systemic immune status. Results showed that compared with the original MulBSTA score, this model has higher efficiency in predicting the risk of severe conversion (AUROC = 0.94937vs.0.8241)(Fig. 2G). The viral pneumonia severity risk prediction model constructed in this study includes indicators of different dimensions such as demographic characteristics, comorbidities, immune function, imaging, and has good predictive ability (AUROC = 0.94937vs.0.8241). Hence, it may help control the risk of severe conversion of viral pneumonia and may become a screening tool for severe viral pneumonia. This study also has some limitations. The retrospective single-center design led to missing partial data and inevitable bias.

## Conclusion

We constructed a risk score incorporating eight previously identified parameters routinely used in hospitals that are highly predictive of severe disease in viral pneumonia. Hospitalized patients were stratified according to the level of risk of critical illness to guide further clinical decisions.

### Supplementary Information

Supplementary Material 1.

Supplementary Material 2.

Supplementary Material 3.

Supplementary material 4.

Supplementary Material 5.

Supplementary material 6.

## Data Availability

No datasets were generated or analysed during the current study.
