# Supplementary figures and images for: Predicting the potentially exacerbation of severe viral pneumonia in hospital by MuLBSTA score joint CD4 + and CD8 +T cell counts: construction and verification of risk warning model
Source: BMC Pulm Med. 2024 May 29;24:261. doi: 10.1186/s12890-024-03073-y (PMC11137986; doi:10.1186/s12890-024-03073-y)

Supplement Figure 1: Study flowchart. RT-PCR: reverse-transcription polymerase chain reaction.


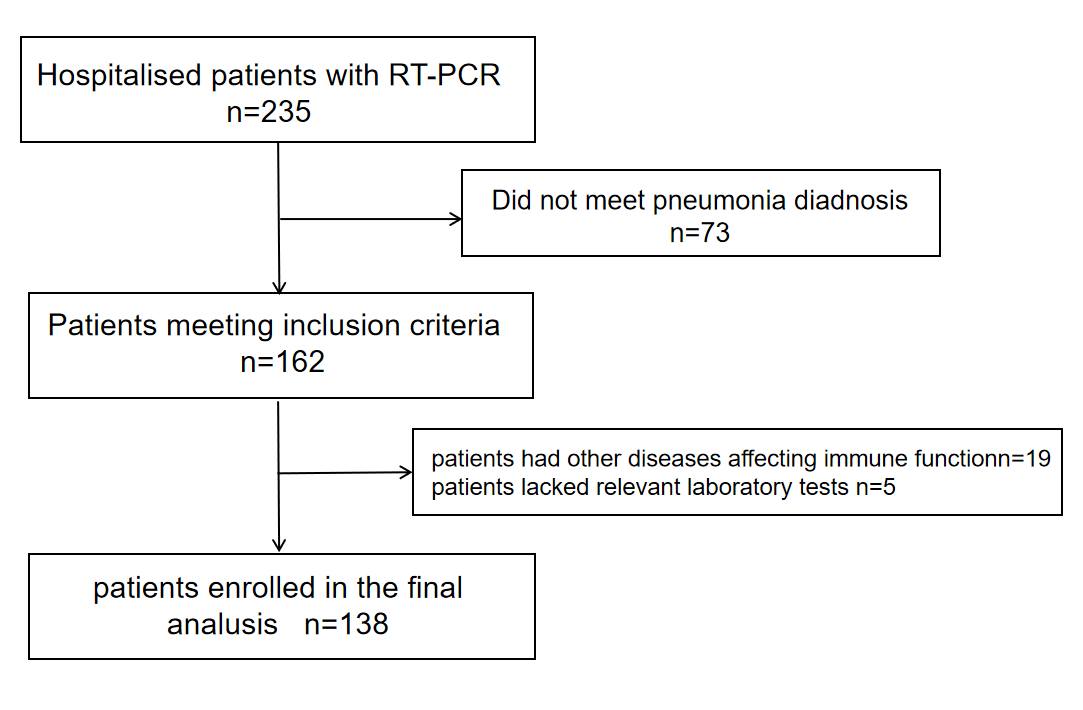

Supplement: Supplementary file 1 — Supplementary Material 1. [file 12890_2024_3073_MOESM1_ESM.docx]
