# Supplementary material for: Predicting the potentially exacerbation of severe viral pneumonia in hospital by MuLBSTA score joint CD4 + and CD8 +T cell counts: construction and verification of risk warning model
Source: BMC Pulm Med. 2024 May 29;24:261. doi: 10.1186/s12890-024-03073-y (PMC11137986; doi:10.1186/s12890-024-03073-y)
Supplement: Supplementary file 3 — Supplementary Material 3. [file 12890_2024_3073_MOESM3_ESM.docx]

supplement table1: Viral pneumonia virus species

| virus species of Viral pneumonia | number |
| --- | --- |
| novel coronavirus | 54 |
| human herpes virus | 41 |
| influenza virus | 11 |
| rhinovirus | 9 |
| human parainfluenza virus | 7 |
| Human adenovirus | 8 |
| enterovirus | 2 |
| respiratory syncytial virus | 2 |
| human metapneumovirus | 2 |
| Bocavirus | 1 |
| Human parvovirus | 1 |
