# Supplementary material for: Predicting the potentially exacerbation of severe viral pneumonia in hospital by MuLBSTA score joint CD4 + and CD8 +T cell counts: construction and verification of risk warning model
Source: BMC Pulm Med. 2024 May 29;24:261. doi: 10.1186/s12890-024-03073-y (PMC11137986; doi:10.1186/s12890-024-03073-y)
Supplement: Supplementary file 4 — Supplementary material 4. [file 12890_2024_3073_MOESM4_ESM.docx]

Supplemental table2 :Types of bacterial infection

| Types of bacterial infection | number |
| --- | --- |
| Candida albicans | 1 |
| Acinetobacter baumannii | 9 |
| enterococcus faecalis | 3 |
| escherichia coli | 7 |
| Haemophillus influenzae | 3 |
| Klebsiella pneumoniae | 10 |
| mycoplasma | 1 |
| Pneumocystis carinii | 2 |
| aspergillus fumligatus | 2 |
| pseudomonas aeruginosa | 5 |
| streptococcus constellatus | 2 |
| streptococcus pneumoniae | 3 |
| hemolytic streptococcus | 2 |

Positive bacterial cultures from blood or sputum specimens were used as a criterion for co-infection with bacteria
