# Supplementary material for: Predicting the potentially exacerbation of severe viral pneumonia in hospital by MuLBSTA score joint CD4 + and CD8 +T cell counts: construction and verification of risk warning model
Source: BMC Pulm Med. 2024 May 29;24:261. doi: 10.1186/s12890-024-03073-y (PMC11137986; doi:10.1186/s12890-024-03073-y)
Supplement: Supplementary file 5 — Supplementary Material 5. [file 12890_2024_3073_MOESM5_ESM.docx]

Supplement Table3. Coefficient estimates were analyzed by LASSO regression

|  | Prediction value |
| --- | --- |
| (Intercept) | -0.0876 |
| age | 0.0045 |
| gender | . |
| Co infection | 0.1087 |
| CD4 | -0.0002 |
| CD8 | . |
| CD4+/CD8+_ratio | -0.0152 |
| lymphocyte | . |
| Multi lobe | 0.1030 |
| smoking | 0.0045 |
| hypertension | 0.0618 |
| ICU admission days | 0.0360 |
| Hospital admission days | 0.0011 |
