# Supplementary material for: Predicting the potentially exacerbation of severe viral pneumonia in hospital by MuLBSTA score joint CD4 + and CD8 +T cell counts: construction and verification of risk warning model
Source: BMC Pulm Med. 2024 May 29;24:261. doi: 10.1186/s12890-024-03073-y (PMC11137986; doi:10.1186/s12890-024-03073-y)
Supplement: Supplementary file 6 — Supplementary material 6. [file 12890_2024_3073_MOESM6_ESM.docx]

Supplement table4：Results Table of Logistic multivariate regression coefficients；

| variable | coefficien | [standard deviation](javascript:;) | 95%CI | P |
| --- | --- | --- | --- | --- |
|  | -6.5640 | 3.4930 | -14.99~-0.63 | 0.0060 |
| age | 0.0405 | 0.0446 | -0.04~0.15 | 0.0363 |
| Co infection (Yes) | 0.5107 | 1.1700 | -1.93~2.88 | 0.0363 |
| CD4 | -0.0012 | 0.0038 | -0.01~0.01 | 0.0074 |
| CD4+/CD8+_ratio | -1.2800 | 1.1890 | -3.98~0.9 | 0.0282 |
| Multi lobe | 1.9370 | 1.1450 | -0.24~4.52 | 0.0091 |
| Smoking (Once) | 1.8390 | 1.5630 | -1.13~5.45 | 0.1239 |
| Smoking | 0.4606 | 1.1950 | -2.07~2.92 | 0.0400 |
| Hypertension | 0.0154 | 1.2520 | -2.73~2.44 | 0.0190 |
| ICU admission days | 8.2460 | 12.2618 | 2.23~12.61 | 0.194 |
| Hospital admission days | 0.1355 | 0.0801 | 0~0.33 | 0.0091 |
